# Production and Partial Characterization of Cellulases from *Trichoderma* sp. IS-05 Isolated from Sandy Coastal Plains of Northeast Brazil

**DOI:** 10.4061/2011/167248

**Published:** 2011-08-29

**Authors:** Jackeline Pereira Andrade, Aline Simões da Rocha Bispo, Phellippe Arthur Santos Marbach, Rodrigo Pires do Nascimento

**Affiliations:** Centro de Ciências Agrárias, Ambientais e Biológicas (CCAAB), Universidade Federal do Recôncavo da Bahia (UFRB), 44380-000 Cruz das Almas, BA, Brazil

## Abstract

This study evaluated the production of cellulolytic enzymes by *Trichoderma* sp. IS-05 strain, isolated from sand dunes, according to its ability to grow on cellulose as carbon source. Wheat bran was tested as the carbon source and peptone tested as the nitrogen source. Different concentrations of carbon and nitrogen were tested using a factorial design to identify optimal cellulase activity production. The results showed that media containing wheat bran 4.0% (w/v) and peptone 0.25% (w/v) lead to the highest production, 564.0 U L^−1^ of cellulase, obtained after 2 days of fermentation. The pH and temperature profile showed optimal activity at pH 3.0 and 60°C. As for thermostability, the cellulase was most tolerant at 60°C, retaining more than 59.6% of maximal activity even after 4 hours of incubation. The combination of acid pH, high temperature tolerance, and production of cellulase from agro-industrial residues by *Trichoderma* sp. IS-05 offers possibilities condition for the biomass hydrolysis process to produce bioethanol.

## 1. Introduction

Cellulosic material is the most abundant renewable carbon source in the world. It is a linear polymer of 8000–12000 glucose units linked together by *β*-1,4-glycosidic bonds, is a major component of plant biomass, and is naturally degraded by cellulolytic fungi and bacteria. Cellulose hydrolyses to glucose, which can then be used for production of ethanol [1], organic acids [2], and other chemicals [3]. This hydrolysis is carried out via the synergic action of three cellulolytic enzymes: endo-*β*-D-glucanase (EC 3.2.1.4), exo-*β*-D-glucanase (EC 3.2.1.91), and *β*-glucosidase (EC 3.2.1.21) [4, 5]. 

Lignocellulosic residues from agriculture and forestry have potential as cheap and renewable feedstocks for the large-scale production of fuels and chemicals. The biodegradation of cellulose to soluble sugar is a process which is only possible after the action of complex cellulolytic produced by cellulolytic microorganisms. In recent years, increased scientific attention has been given to this process due to its environmental and economic significance [6]. Wheat bran is one of the most common agroindustrial residues used as raw material for various processes and products. Industrial wheat bran usually accounts for 14–19% of the grain and comprises the outer coverings, the aleuronic layer, and the remnants of the starchy endosperm. It consists mainly of starch, (glucurono)arabinoxylans, cellulose, *β*-glucan, protein, and lignin [5]. 

Significant research efforts have been invested in evaluating and understanding the enzymatic hydrolysis of lignocellulosic substrates by cellulases produced by species of the fungus *Trichoderma *[7–10]. Commercial products of various *Trichoderma *isolates have long been available for cereal foods, brewing, and fruit and vegetable processing and have also been widely evaluated and applied for bioethanol production processes [11].

Studies dealing with cellulase production by fungi using low-cost residues are abundant in the literature. *Trichoderma* species, one of a wide range of cellulase-producing organisms, is ubiquitous in Brazil, being commonly found in lignocellulosic residues and soils. The present work reports on cellulolytic enzyme production by a strain, identified in our laboratory as IS-05 that was isolated from sand dunes on the coastal plains, in Guaibim, BA, Brazil. An experimental design was carried out to study cellulase production using wheat bran (WB) as the carbon source and peptone as the nitrogen source. Crude enzyme preparations (culture supernatants) were used to perform preliminary studies for the temperature and pH activity profile and effect of metal ions on enzyme activity.

## 2. Materials and Methods

### 2.1. Isolation, Selection, and Inoculums Preparation of the Fungal Strain

*Trichoderma *sp. IS-05 was collected from the sand dunes at Guaibim beach, BA, Brazil and then isolated and identified by morphological aspects [12]. The crude sample was diluted in 0.85% (*w/v*) saline solution (1 : 10), stirred at 150 rev.min^−1^ for 45 min and serial diluted. Dilutions were plated on microcrystalline cellulose-salt mineral medium agar consisting of (g L^−1^) 2.6 (NH_4_)_2_SO_4_; 2.0 NaCl; 3.0 KH_2_PO_4_; 6.0 K_2_HPO_4_; 0.2 MgSO_4_·7H_2_O; 0.02 CaCl_2_·2H_2_O; 10.0 microcrystalline cellulose (Sigma); 15.0 agar; 1.0 mL trace solution (0.64 g CuSO_4_·5H_2_O; 0.15 g ZnSO_4_·7H_2_O; 0.11 g FeSO_4_·7H_2_O; 0.79 g MnCl_2_·4H_2_O; 100 mL distilled water). The inoculated plates were incubated for 10 days at 30°C, and the grown fungi were cultivated in pure culture. 

The cellulase-producing capacity of the strain was carried out using filter paper cellulose, in Mandel's media [13], pH 6.0 (g L^−1^): 2.0 K_2_HPO_4_, 1.4 (NH_4_)_2_SO_4_, 0.3 MgSO_4_·7H_2_O, 0.3 CaCl_2_, 0.005 FeSO_4_·7H_2_O, 0.00156 MnSO_4_·H_2_O, 0.0014 ZnSO_4_·7H_2_O, 0.002 CoCl_2_, 3.0 yeast extract, supplemented with 0.6 g of Whatman Filter Paper Grade N°1 (1.0 × 1.0 cm). The flasks were incubated at 28°C, stirred at 180 rev.min^−1^ for 7 days and daily aliquots of 2.0 mL were collected, filtered, and the cellulase activity was analyzed as described below.

 For inoculums production, fungal spores of culture grown on Potato Dextrose Agar (PDA) at 28°C for 15 days, were harvested in sterile saline solution (0.85% *w/v*), as described by Hopwood et al. [14]. Spores were maintained in 20% (*v/v*) glycerol at −20°C.

### 2.2. Cellulase Production Using Experimental Design

Cellulase activity was measured after cultivation in 250 mL Erlenmeyer flasks containing 50 mL of mineral salts solution [5], pH 6.5 (g L^−1^, KH_2_PO_4_, 3.0; (NH_4_)_2_SO_4_, 2.6; K_2_HPO_4_, 6.0; MgSO_4_·7H_2_O, 0.2; NaCl, 2.0 and CaCl_2_, 0.002) supplemented with a trace element solution (g L^−1^, FeSO_4_·7H_2_O, 1.1; MnCl_2_·4H_2_O, 7.9; ZnSO_4_·7H_2_O, 1.5 and CuSO_4_·5H_2_O, 6.4). Wheat bran (WB) was added as the main carbon source, and peptone was used as the main nitrogen source. The initial pH of the medium was adjusted to 5.0. Culture medium was inoculated with 50 *μ*L of spore suspension (6.22 × 10^9^ spores mL^−1^), incubated at 28°C, and shaken for 6 days. At periodical intervals (24 hours), flasks were collected, its whole content centrifuged (2500 g for 10 min), filtered, and the supernatants were tested in cellulase activity assays. The supernatants were preserved at −20°C. Results were presented as an average of duplicates.

Optimization of the concentration of WB (C source) and peptone (N source) at 200 rev.min^−1^ was carried out by employing a response surface methodology. As the dependent variable we used the cellulase activity (UL^−1^) and the independent variables we used the C source (WB) and N source (peptone) concentrations. A 2^2^ full factorial central composite rotational design (CCRD) was used to generate 11 run combinations as shown in Table 1 [15]. This design is represented by a second-order polynomial regression model, (1), to generate contour plots:

(1) $$Y=b_{0}+b_{1}X_{1}+b_{2}X_{2}+b_{12}X_{1}X_{2}+b_{11}X_{1}^{2}+b_{22}X_{2}^{2},$$

where *Y *is the predicted response (cellulase activity); *X*_1_, *X*_2_ and *X*_3_ the coded forms of the input variables (WB and peptone, resp.); *b*_0_ a constant; *b*_1_ and *b*_2_ the linear coefficients; *b*_12_ a cross-product coefficient; *b*_11_ and *b*_22_ the quadratic coefficients. The test factors were coded according to the following regression equation:

(2) $$x_{i}=\frac{\left(X_{i}-\;X_{0}\right)}{\Delta X_{i}},$$

where *x*_*i*_ is the coded value and *X*_*i*_ the actual value of the independent variable, *X*_0_ is the actual value at the center point and Δ*X*_*i*_ is the step change value.

 ANOVA (analysis of variance) was used to estimate the statistical parameters. The significance of the regression coefficients was determined by the Student's *t*-test; the second-order model equation was determined by Fisher's test. The variance explained by the model is given by the multiple coefficient of determination, *R* ^2^. STATISTICA (version 7.0) software from StatSoft Inc. was used for the regression and graphical analysis.

The same medium used in the preliminary tests, supplemented with different combinations of WB as the carbon source and peptone as the nitrogen source, was used for the experimental design (Table 1). Conditions for the inoculation, incubation, and filtration of the supernatant were used as preliminary studies on cellulose production (data not show).

Based on the CCRD experiment, a validation was carried out using, in triplicate, the conditions suggested by the model. The concentration of peptone was fixed at 0.25% (*w/v*) and the WB concentrations used were 4.2%, 4.4%, 4.6%, and 4.8% (*w/v*), generating 4 validation assays. The system was incubated at 28°C/200 rev min^−1^ for 2 days. The supernatant was collected as described above.

### 2.3. Enzyme Assays

Cellulase (filter paper activity) was assayed by measuring the release of reducing sugars in a reaction mixture containing Whatman N°1 filter paper (1.0 cm × 6.0 cm *≅* 50 mg) as substrate in 50 mM sodium citrate buffer (pH 4.8) at 50°C after 30 min. One unit (U) of cellulase activity corresponded to 1 *μ*mol of glucose equivalent released per minute under the assay conditions [16]. Reducing sugars were assayed by the dinitrosalicylic acid (DNS) method [17].

All assays were conducted in duplicates, and results expressed as average values. Variations in the multiple assays were <5%.

### 2.4. Partial Crude Enzyme Characterization

Temperature profile for cellulase activity was determined by assaying activity at different reaction temperatures (20 to 80°C) in 50 mM sodium citrate buffer (pH 4.8). In the same way, cellulase activity was assayed in different reaction buffers 50 mM (glycine-HCl for pH 2.0–3.0; sodium citrate for pH 3.0–6.0; citrate phosphate for pH 6.0-7.0; phosphate for pH 7.0-8.0; Tris HCl for pH 8.0–10.0) at 60°C to determine the effect of pH on activity. For comparison, some tests (pH and temperature effect) were carried out using the commercial cellulase CAREZYME by Novozyme. 

To determine the thermal stability, the crude supernatant was incubated at 60°C and the residual cellulase activity was measured at various time periods (0.5, 1, 2, 4, and 6 h). 

The influence of various metal ions on cellulase activity was evaluated with enzymatic assay at pH 3.0 and 60°C after addition of each ion (potassium, barium, iron, calcium, sodium, cobalt and mercury in the chloride form and zinc, manganese, copper, magnesium in the sulfate form) at 10 mM final concentration [5]. The influence of ethylenediamine tetraacetic acid (EDTA) was also tested at the same concentration.

## 3. Results and Discussion

The fungal strain IS-05, identified as *Trichoderma* sp., was capable of degrading microcrystalline cellulose in a solid plate medium and was therefore selected for further studies. In the present work, we investigated cellulase production by *Trichoderma* sp. using agroindustrial by-products as substrates. In a preliminary experiment, 54 fungal strains isolated from Guaibim sand dunes were grown in submerged fermentation in Mandel's medium supplemented with Whatman N°1 Filter Paper as the sole carbon source (data not show). The *Trichoderma* sp. strain IS-05 was selected as a cellulolytic-promising strain for further fermentation studies. Table 1 presents the observed and predicted results, obtained after cultivation for 2 days. Cellulase activity varied from 8.0 to 564.0 U L^−1^. The best result was obtained on Run 4, with WB and peptone concentrations of 4.0% (*w/v*) and 0.25% (*w/v*), respectively. When the concentrations of WB were 4.0% and 3.0% (*w/v*), the cellulase activity was 447.0 and 465.1 U L^−1^, respectively, for Runs 2 and 7. Generally, best results of enzyme production were obtained for high concentrations of WB (Runs 2, 4, 6 and 7) together with low concentrations of the N source. 

The model was tested for adequacy by the analysis of variance (ANOVA). The computed *F*-value (14.43) indicates that the model was significant at a high confidence level. The probability *P *value was also very low (<0.01) indicating the significance of the model (Table 2). The coefficient of variation (*R* ^2^ = 0.86) also indicates a very good correlation between the experimentally observed and predicted values. The mathematical model representing the cellulase activity (*Y*) for the combination WB (*X_1_*) + peptone (*X_2_*) in the experimental region studied can be expressed by

(3) $$Y=285.14+140.41X_{1}-43.51X_{1}^{2}+40.97X_{2}^{2}.$$

**Figure 1:**
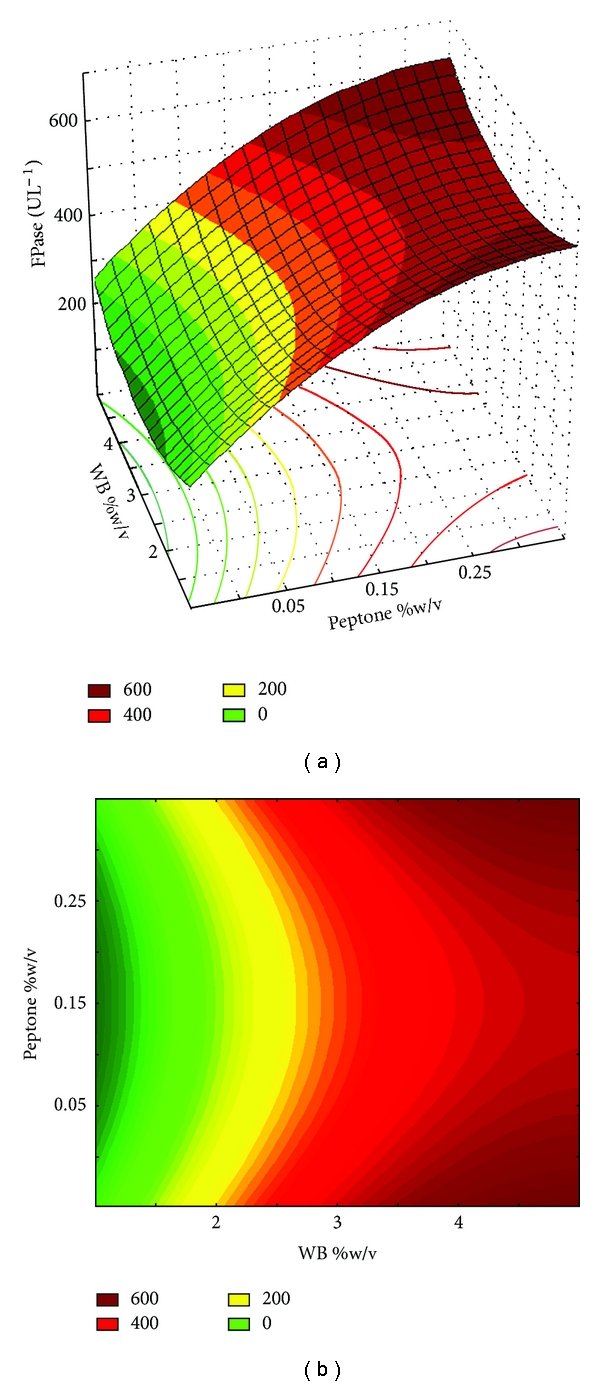
Response surface (a) and contour curve (b) on cellulase production by *Trichoderma *sp. IS-05 using WB and peptone concentrations as independent variables. The full factorial central composite design (2^2^) used the response surface methodology to predict the best point for cellulase production.

Although the concentration of the independent variable WB had a significant effect on the cellulase production, interactions between WB and peptone did not (*P *> 0.1). The regression analysis for the experiment using the combination WB + peptone, (3) shows the significant coefficients of the full second-order polynomial model of cellulase production, determined by Student's *t*-test and *P* values. The resulting surface response plots and contour curve showing the effect of substrate concentration (WB and peptone) on the cellulase production by *Trichoderma *sp. IS-05 are presented in Figures 1(a) and 1(b). 

The validation of the mathematical model used based on the CCRD experiments confirmed the maximal values for cellulase obtained, from 592.5 to 1224.0 U L^−1^ (4.8% and 4.4% (*w/v*) WB, resp.) supplemented with 0.25% (*w/v*) peptone, after 2 days fermentation. 

According to the literature, it is well known that fungi, especially *Trichoderma* and *Aspergillus*, are able to degrade agroindustrial residues through lignocellulolytic enzymes, including cellulases [18–20]. Kovács et al. [19] studied the production of cellulase, among other enzymes by *Trichoderma reesei* RUT-C30 and other *Trichoderma* sp. mutant strains grown with pretreated willow (15 g L^−1^) and cellulose powder Sigmacell type 20 (10 g L^−1^). The highest cellulase activities observed were 620.0 U L^−1^ (pretreated willow) and 1090.0 U L^−1^ (cellulose powder Sigmacell type 20) after 3 days fermentation. Wen et al. [20] reported the effect of different dairy manure concentrations on cellulase production by *Trichoderma reesei* RUT-C30. The best result was 1200 U L^−1^ when 13 g L^−1^ of dairy manure was used. Jiang et al. [21] observed a quite similar cellulase activity (880 U L^−1^) with a new isolate of *Trichoderma viride* strain using phosphoric acid swollen cellulose as carbon source. Our group has investigated various *Trichoderma* and *Aspergillus* strains using agroindustrial residues in order to produce lignocellulose degradation enzymes, including endoglucanases. The cellulase titers obtained, using WB (1224.0 U L^−1^) as the carbon source and peptone as the nitrogen source, under submerged culture conditions, in this study were higher than the cellulase titers of 46.0 U L^−1^ obtained by Grigorevski-Lima et al. [6] also using WB with *A. fumigatus* FBSPE-05. Pothiraj et al. [22] observed maximum values for carboxymethylcellulase (CMCase) and filter paper activity (cellulase) of 120 U L^−1^ and 40 U L^−1^, respectively, after 8 days fermentation for *A. niger*, using cassava waste as the carbon source. For *A. terreus*, for the same fermentation period, Pothiraj et al. [22] observed lower values for CMCase (100 U L^−1^) but the same values for cellulase (40 U L^−1^). Considering two days fermentation, the *Trichoderma* sp. strain IS-05 (1224.0 U L^−1^) produces 26.61 times more cellulase than *A. fumigatus* (46 U L^−1^) [6] and 1.39 times more than *T. viride* (880 U L^−1^) [21].

**Figure 2:**
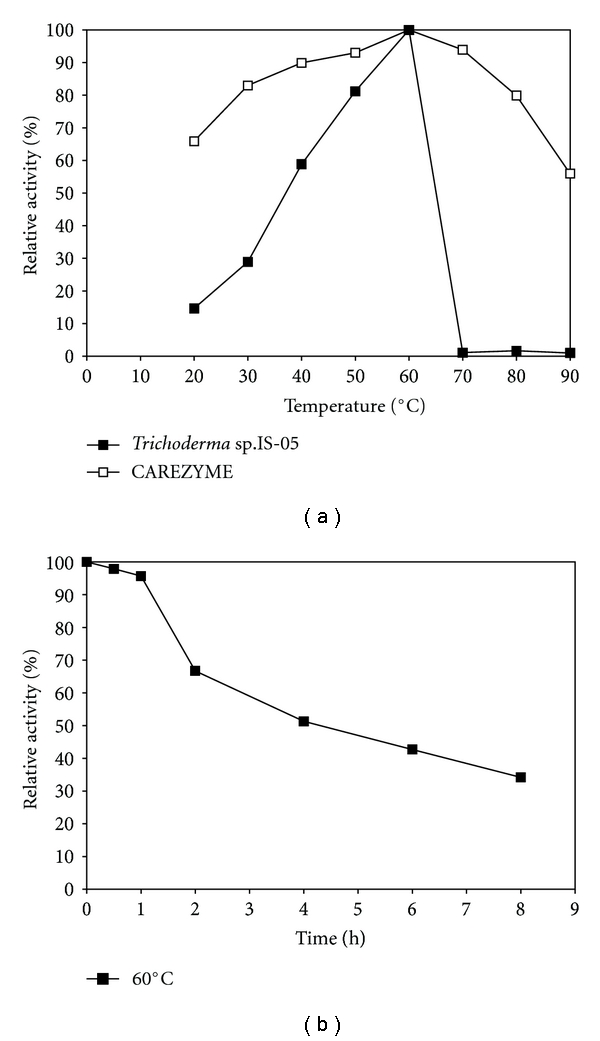
Effect of temperature (a) (■) and thermal stability at 60°C (b) on cellulase activity (pH 4.8) produced by* Trichoderma *sp. IS-05 grown on 4.4% (*w/v*) WB, 0.25% (*w/v*) peptone, and commercial enzyme CAREZYME (□). Residual activity is expressed as a percentage of the original activity. Error bars represent the standard deviation of each experimental point (*n* = 2) (100% residual activity = 1036.9 U L^−1^).

Cellulases present in the crude supernatant obtained from *Trichoderma* sp. strain IS-05 grown in 4.2% (*w/v*) WB and 0.25% (*w/v*) peptone in submerged fermentation showed maximal activity at 60°C (Figure 2(a)), and activity values of approximately 81% were still detected at 50°C. Other studies using *Aspergillus niger* [18] have shown a residual activity of around 100% for cellulase activity at temperatures between 50° and 60°C, very similar to our results. Crude enzyme from *Trichoderma *sp. strain IS-05 was able to retain 59.6% residual activity at 60°C for 4 h; the half-life of crude enzyme being 5 h at 60°C (Figure 2(b)). Half-lives of 8 h at 60°C or 1 h at 70°C have been cited in the literature for some *Aspergillus niger* [18]. Our results strongly suggest that the cellulases in this supernatant seem to be thermophilic, which are considered ideal for many biotechnological processes. 

CAREZYME is a commercial enzyme preparation produced by submerged fermentation of a genetically modified *Aspergillus* microorganism. Optimum temperature for CAREZYME and *Trichoderma* sp. IS-05 enzyme preparations were the same (60°C). However, CAREZYME was able to retain over 70% of relative activity in the range between 30 and 80°C and retain 50% of the maximum activity even at 100°C, while the crude supernatant of *Trichoderma* sp. IS-05 was unable to retain any enzyme activity at temperatures above 70°C (Figure 2(a)).

**Figure 3:**
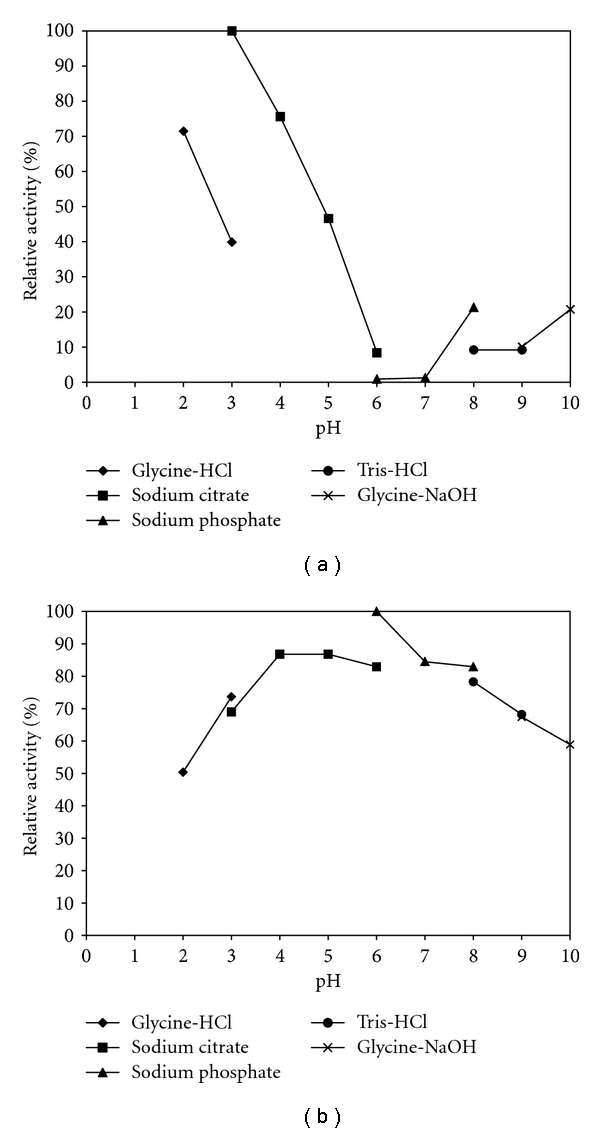
Effect of pH on cellulase activity at 60°C produced by* Trichoderma *sp. IS-05 (a) grown on 4.4% (*w/v*) WB, 0.25% (*w/v*) peptone, and commercial enzyme CAREZYME (b). The ionic strength for all buffers was 50 mM: glycine-HCl (*◆*); sodium citrate (■); sodium phosphate (▴); Tris-HCl (*∙*); glycine-NaOH (-x-). Residual activity is expressed as a percentage of the original activity. Error bars represent the standard deviation of each experimental point (*n* = 2) (100% residual activity = 1079.7 U L^−1^).

The pH profiles (Figure 3) have shown more than 80% activity in the acidic pH range (2.0 to 4.0), with optimal activity occurring at pH 3.0. Values in the neutral range (6.5 to 7.5) of pH were very low, around 9% of residual activity at these pH values. However, in the alkali range, a new peak (21%) of cellulase activity at pH 10.0 was observed, suggesting the possibility of two cellulases. This biochemical characteristic could be very interesting for processes that require acidic conditions. There are few reports in the literature about cellulase activity in an acidic pH range. Nascimento et al. [23] have shown a pH activity profile within the range 2.0–5.0, with maximum activity observed at pH 4.0. CMCase activity in the acid pH range was also detected by Grigorevski-Lima et al. [6] for *Aspergillus fumigatus*. 

The pH studies comparing two preparations have shown different pH profile patterns with major differences in optimal activity, which were pH 3.0 for *Trichoderma* sp. IS-05 supernatant (Figure 3(a)) and pH 6.0 for CAREZYME (Figure 3(b)). Differences in the results concerning some of the pH tested using different buffers were observed, especially in pH 3.0 for *Trichoderma* sp. IS-05 and pH 6.0 in CAREZYME. In fact, according to the buffers used to maintain each pH value, different activities were observed in same pH value. At pH 3.0, for *Trichoderma* sp. IS-05 the differences suggest a greater affinity of the crude enzymatic extract in sodium citrate buffer. The same can be said about results at pH 6.0 for the commercial enzyme. In fact, a similar result has been reported in the literature [5, 6, 23, 24].

Results of cellulase activity in the presence of metal ions are shown in Table 3. All ions tested had significant effect on cellulase activity. A considerable decrease (>80% inhibition) in activity was observed in the presence of Co^+^, Cu^2+^, and Mn^2+^. These ions are commonly cited in the literature as inhibitors for several microbial cellulases [25–27]. Activity is probably inhibited through the attack of certain groups at the active site of the enzyme, for example, the thiol groups, leading to inactivation [25]. According to these results, these ions must be avoided in future cultivations for a high cellulase production.

## 4. Conclusions

The fungi strain *Trichoderma *sp. IS-05 used in this study was able to grow and produce good levels of cellulase using wheat bran and peptone as the sole sources of C and N. The maximum cellulase activity detected was of 1224 U L^−1^, on the second day of cultivation, when a mineral medium was supplemented with peptone 0.25% (*w/v*) and WB 4.4% (*w/v*). These results were obtained after using the validation of factorial experimental design for optimization. The validation of experimental design resulted in a 2.17-fold improvement on cellulase production when compared with first results on cellulase matrix optimization in CCRD. The optimum pH and temperature of the crude extract were 3.0 and 60°C, respectively. Considering the design of the medium, and the high titers obtained for enzymatic activity, the results obtained indicate a possible use for these crude enzymatic extracts in biotechnology processes, especially for lignocellulosic biomass hydrolysis without contamination (pH 3.0/60°C) for generating reducing sugars for bioethanol production.

## Figures and Tables

**Table 1:** Values of independent variables (WB concentration *X_1_* and peptone concentration *X_2_*, resp.) used in CCRD, showing the values observed and predicted by the mathematical model for cellulase production by *Trichoderma *sp. IS-05.

Run	Coded setting levels		Actual levels (% *w/v*)		Cellulase activity (U L^−1^)	
	*X*_1_	*X*_2_	*X*_1_	*X*_2_	O	P
1	−1	−1	2.0	0.05	8.2	102.2
2	+1	−1	4.0	0.05	447.3	463.0
3	−1	+1	2.0	0.25	83.6	102.2
4	+1	+1	4.0	0.25	563.6	463.1
5	−1.41	0	1.59	0.15	21.3	−55.7
6	+1.41	0	4.41	0.15	389.9	453.2
7	0	−1.41	3.0	0.009	465.3	366.6
8	0	+1.41	3.0	0.291	281.8	366.7
9	0	0	3.0	0.15	275.3	285.1
10	0	0	3.0	0.15	288.4	285.1
11	0	0	3.0	0.15	291.6	285.1

Results are the mean of two experiments, O = observed; P = predict.

**Table 2:** Statistical analysis of variance (ANOVA) for the model of cellulase production at different levels of concentration of WB and peptone.

Source of variations	Sum of squares	Degrees of freedom	Mean square	*F*-value	*P* value*
Regression	287810.6	3	95936.9	14.4	0.007
Residue	46549.1	7	6649.9		
Total SS	334359.7	10			

*Statistically significant at 90% of confidence level. *R* ^2^ = 0.86.

**Table 3:** Effect of different ions on cellulase activity. Enzyme was produced by* Trichoderma *sp. IS-05 grown on 4.4% (*w/v*) WB and 0.25% (*w/v*) peptone.

Ion^a^	Relative activity (%)^b^
Control (no addition)	100.0
EDTA	42.4
Mg^2+^	36.6
Zn^2+^	48.4
Co^+^	14.1
K^+^	63.7
Cu^2+^	11.7
Na^+^	46.6
Ba^2+^	33.9
Mn^2+^	18.4
Ca^2+^	44.0
Hg^+^	31.4
Fe^2+^	30.7

^a^The final concentration in the reaction mixture was 10 mM.

^b^Relative activity is expressed as a percentage of control (100% of enzyme activity = 1224.0 U L^−1^).
